# What roles do accredited drug dispensing outlets in Tanzania play in facilitating access to antimicrobials? Results of a multi-method analysis

**DOI:** 10.1186/s13756-015-0075-2

**Published:** 2015-08-20

**Authors:** John C Chalker, Catherine Vialle-Valentin, Jafary Liana, Romuald Mbwasi, Innocent A Semali, Bernard Kihiyo, Elizabeth Shekalaghe, Angel Dillip, Suleiman Kimatta, Richard Valimba, Martha Embrey, Rachel Lieber, Edmund Rutta, Keith Johnson, Dennis Ross-Degnan

**Affiliations:** Center for Pharmaceutical Management, Management Science for Health, 4301 North Fairfax Drive, Arlington, VA 22203 USA; Drug Policy Research Group, Department of Population Medicine, Harvard Pilgrim Health Care Institute, 133 Brookline Avenue, 6th Floor, Boston, MA 02215 USA; Management Science for Health, Dar es Salam, Tanzania; Apotheker Consultancy (T) Ltd;, St. Johns University of Dodoma, Dodoma, Tanzania; Muhimbili University of Health and Allied Sciences, P.O. Box 65015, Dar es Salaam, Tanzania; Tanzania Consumer Advocacy Society (TCAS), Dar es Salaam, Tanzania; Pharmacy Council of Tanzania, P.O. Box 31818, Dar es Salaam, Tanzania; Ifakara Health Institute, Plot 463, Kiko Avenue Mikocheni, P.O. Box 78 373, Dar es Salaam, Tanzania

**Keywords:** Tanzania, Antimicrobial resistance, Medicine shops, ADDOs, Antibiotics

## Abstract

**Background:**

People in low-income countries purchase a high proportion of antimicrobials from retail drug shops, both with and without a prescription. Tanzania’s accredited drug dispensing outlet (ADDO) program includes dispenser training, enforcement of standards, and the legal right to sell selected antimicrobials. We assessed the role of ADDOs in facilitating access to antimicrobials.

**Methods:**

We purposively chose four regions, randomly selected three districts and five wards per district. Study methods included interviews at 1200 households regarding care-seeking for acute illness and knowledge about antimicrobials; mystery shoppers visiting 306 ADDOs posing as a caregiver of a child with 1) pneumonia, 2) mild acute respiratory infection (ARI), or 3) a runny nose and request for co-trimoxazole; and audits of antimicrobial availability and prices at 84 public health facilities (PHFs) and 96 ADDOs.

**Results:**

Four hundred sixty seven (76 %) members from 367 (77 %) households had recently sought care outside the home for acute illness; 128 had purchased antimicrobials, of which 61 % had been recommended by a doctor or nurse and 32 % by an ADDO dispenser. Only 29 % obtained the antimicrobial at a PHF, whereas, 48 % purchased them at an ADDO. Most thought that ADDOs are convenient place for care, usually have needed medicines, and have high quality services and products, contrasting with 66 % who reported dissatisfaction with PHF waiting times and 56 % with medicine availability. One-third (34 %) of mystery shoppers presenting the mild ARI scenario were inappropriately sold an antimicrobial and 85 % were sold one on request; encouragingly, 99 % presenting a case of pneumonia received either an antimicrobial, referral to a trained provider, or request to bring the child for examination. Overall, 63 and 60 % of the 15 tracer antimicrobials were in stock in ADDOs and PHFs, respectively; ADDOs had significantly more antimicrobial formulations for children available (83 vs. 51 %). Of 369 records of antimicrobial sales in 47 ADDOs, 63 % were dispensed on prescription.

**Conclusion:**

ADDOs have increased access to antimicrobials in Tanzania. Community members see them as integral to the health system. Antimicrobials are overused due to poor ADDO dispensing, poor PHF prescribing, and inappropriate public demand. Multi-pronged interventions are needed to address all determinants.

**Electronic supplementary material:**

The online version of this article (doi:10.1186/s13756-015-0075-2) contains supplementary material, which is available to authorized users.

## Background

A recent World Health Organization (WHO) report points out that “very high rates of resistance have been observed in bacteria that cause common health-care associated infections in all WHO regions” [1]. Factors that contribute to antimicrobial resistance include unnecessary overuse and under dosing of antimicrobials [2]. In low- and middle-income countries, only 70 % of pneumonia cases receive an appropriate antimicrobial and about half of all acute viral upper respiratory tract infection and viral diarrhea cases receive antimicrobials inappropriately [3].

In low-resource countries, consumers often seek care and purchase medicines, including antimicrobials, at small retail drug shops [4, 5]. In some rural settings, up to 80 % of people seek care at such shops because few licensed pharmacies or pharmacists exist outside of cities [6, 7] Other reasons for drug shop popularity include shorter waiting time, nearness to home, and more reliable drug availability compared with public health facilities (PHFs) [8]. In a review of 51 low- and middle-income countries the cost of medicines accounted for 100 % of health care costs for 41 to 56 % of households; 40 % of poor households used savings, borrowed money, or sold assets to pay for care [9].

In Tanzania, medicines need to be purchased whether they are obtained at public facilities or drug shops, unless the patient has an exempt status, such as the elderly. In the past, unaccredited medicine shops were only allowed to sell over-the-counter drugs; antimicrobials were either sold illegally by the shops or accessed at health facilities where supply problems persisted [10]. To address this situation, the Tanzania government adopted the ADDO program, which was piloted in Ruvuma region in 2003, as national policy. The accredited drug dispensing outlet (ADDO) program combines extensive training, business incentives, authorization to dispense a limited list of antimicrobials and other medicines to treat common conditions, regulatory enforcement of practice standards, and efforts to affect customer demand [11]. After 10 years of the program, Tanzania has about 9000 accredited (or nearly accredited) shops distributed throughout the country in all regions and districts, and over 19,000 trained dispensers. With a population of 45 million, this translates to approximately one ADDO per 5000 people. Previous evaluations of the program have shown that ADDOs have contributed to improvements in the use of antimicrobials [12, 13].

In this study in 2013, we carried out a multi-component assessment to determine how antimicrobials are currently accessed and used in rural communities in Tanzania, with a particular focus on the role of ADDOs.

## Methods

### Sampling

We purposively selected four regions to be both logistically feasible and to span the range of experience with ADDOs. Morogoro represented the eastern regions and had more than 5 years’ of experience in implementing ADDOs; Tanga represented the northern regions and had 2 years’ of experience of implementing ADDOs; Mbeya represented the relatively high socioeconomic status southern highland regions and had 2 to 3 years’ of experience in implementing ADDOs; and Singida represented the relatively low socioeconomic status central regions and had implemented ADDOs in 2008. At the time of the study, Mbeya had 627 ADDOs, Singida 139, Morogoro 658, and Tanga 274.

Once we selected the four regions, we developed a two-stage cluster sample as recommended by WHO/Medicines Transparency Alliance [14]. Within each region sampled, three districts were randomly selected with probability proportional to population size. A complete list of wards and numbers of ADDOs was compiled for each sampled district. High-density wards included wards with five or more ADDOs, except in Singida’s districts, where fewer ADDOs are registered, so the high-density threshold was lowered to three or more ADDOs. Low-density wards included all other wards with ADDOs. Wards without a registered ADDO were classified in a third category. From these lists, we randomly selected two high-density wards, two low-density wards, and one ward with no ADDOs with probability proportional to the population size in each sampled district, for a total of 60 wards.

All villages in the selected wards with ADDOs were grouped into two strata: those close to an ADDO (within 5 km) or far from the ADDO (more than 5 km). In wards without ADDOs, we grouped all villages into two strata: within 5 km of the ward population center or more than 5 km from the ward population center. We gave each village a serial number that we used to randomly select three villages from the first stratum and one from the second.

We selected five households within each selected village by numbering each household, dividing the number of houses in the village by five and then dividing the list into segments comprising that number of households to obtain a sampling interval. A house was selected randomly in the first segment using the last two numbers of village chairperson’s cell phone number. The other four households in the village were selected systematically from the list using the sampling interval. If no one was available in the selected household, the team left a message with a neighbor to inform them that the team would return later in the day. If the interviewer still did not find the responsible person there, he or she chose the next house in the same direction.

### Household data collection

The survey of 1200 households characterized medicines access, use, and antimicrobial resistance knowledge and perceptions in the community. Interviewers selected the respondent who was the most knowledgeable person about the health of household members and their use of medicines. They asked respondents whether anyone in the household had an acute illness in the previous two weeks; if so, we assessed their perception of the illness severity and whether and where each person had sought advice, care, and medicines for the illness. Interviewers also examined the household’s stock of medicines and asked respondents about their views on the quality of service at ADDOs and PHFs and about their knowledge of antimicrobials.

### Mystery shoppers

To assess ADDO performance we conducted 306 mystery shopper visits at ADDOs in the 60 wards. Using mystery shoppers is a well-documented evaluation method [15] that has been used successfully in Tanzanian ADDOs before [12]. Members of the Tanzania Consumer Advocacy Society from each region were trained to pose as a parent or caregiver of a one-year-old child. To select ADDOs for the sample, we used a similar randomization process as for households; we listed all ADDOs in the ward and chose the required number by systematic random sampling.

Each ADDO received one visit from a mystery shopper acting out one of three scenarios (102 visits for each scenario): pneumonia (child with cough, difficulty breathing, and fast breathing with harsh noise); mild ARI (child with cough and runny nose); mild ARI with a request for an antimicrobial. For the first two scenarios, mystery shoppers presented only symptoms and waited for the ADDO dispenser’s response, while for the third scenario, they described symptoms then explicitly requested Septrin. Septrin© is a brand of cotrimoxazole (sulfamethoxazole-trimethoprim) that is widely used locally, so it was reasonable for shoppers to ask for it by name. We chose co-trimoxazole because at that time, it was the recommended drug for managing pneumonia, and the ADDO dispensers had been trained in its use.

### Facility audits

To examine availability and price of antimicrobials in facilities, we randomly selected three ADDOs in each high-density ward and one ADDO in each low-density ward, for a total of 96 ADDOs. We also randomly selected one to two PHFs (public hospitals, health centers, dispensaries) per high ADDO density ward, one per low density ward, and one per ward with no ADDOs, for a total of 84 PHFs. In each ADDO and PHF, we checked drug stocks and selling prices for a tracer list of 15 antimicrobials. The selection criteria for the tracer antimicrobials were inclusion on both the National Essential Drug List and the authorized list of ADDO medicines. They included different medicine formulations (tablets, capsules, dry syrups, suspensions, and injectable) appropriate for child and adult illnesses. We calculated the medicine price ratio (MPR) for each product by comparing the customer price with the median supplier price in the *International Drug Price Indicator Guide 2012* [16]. We then compared the medians of these ratios for ADDOs and PHFs.

### Data analysis

The interviewers entered data for the household survey directly onto preprogrammed tablets and downloaded it to a database. Data from the three surveys were analyzed with Stata survey commands that use sampling weights to adjust for the two-stage clustered sample structure. Wards were considered the primary sampling units for both facility and household surveys. The four purposively selected regions were treated with equal weights in calculating sample-wide estimates. Population sampling weights were applied in the analysis of the household surveys.

We present household survey data as percentage estimates with 95 % confidence intervals, adjusted by household distance from the closest ADDO, as well as by respondent’s age, gender, and level of education, where appropriate. We analyzed household survey opinions related to health care services delivery in three steps: first, we organized responses into four groups each representing a domain: ADDO services (eight questions), services in public health care facilities (four questions), ADDO services in relation to services in other facilities (four questions), and affordability of medicines (two questions) (Additional file 1: Annex 1 includes a table of these domains). Second, we calculated the rotated principal component factors for each set of questions and generated factor scores representing each of the four domains. Finally, we used univariate and multivariate regression to test the predictive value of region of origin, household size, and respondent’s gender, age, and level of education on each domain score (Table 2 and Additional file 1: Annex 2).

From the facility survey data, we calculated the availability of each antimicrobial in health care facilities, by type of facility and region; availability is presented as percentage estimates with 95 % confidence intervals. The median price ratio (MPR) for each product was calculated as the unit price charged compared to the median supplier price listed in the International Management Sciences for Health Drug Price Indicator Guide 2012 [16]. We used Stata regression survey commands to test the predictive value of region and type of facility on antimicrobial MPRs. We also compared the medians of antimicrobial MPRs between ADDOs and PHFs with a paired *t*-test.

For the mystery shopper visits, we did not use a probability sample and did not weight the data. Comparison of results across scenarios was performed with a chi-square test. Our logistic regression analysis considered mild ARI as the reference situation for comparison of the ADDO dispensers’ behavior in handling the other two scenarios.

### Ethical clearance

We obtained ethical clearance for the study from the Tanzanian Ministry of Health and Social Welfare’s National Institute for Medical Research and the Harvard Pilgrim Health Care Institutional Review Board. Authorities for the selected regions and districts granted permission for the study after being briefed by the Pharmacy Council of Tanzania. We explained the study to all household respondents, who signed consent forms to verify their willingness to participate.

## Results

### Household survey

The research team interviewed representatives from 1185 households with 6384 members. Of these, 614 household members (10 %) from 489 households (41 %) had suffered from an acute illness in the previous two weeks. Of those, 467 (76 %) sought care outside the home. The more severe the condition was perceived, the more likely the sick individuals were to seek external care, varying from 91 % of those who perceived their symptoms as severe to 70 % of those who did not. The most common reported symptoms or diagnoses were malaria (51 %) and common cold (49 %), followed by pain (17 %), thirst/sweating (17 %), and watery diarrhea (5 %).

The 467 people with an acute illness sought care in 545 locations; 238 (44 %) went to a public health center or district hospital, 180 (33 %) went to an ADDO, and 106 (19 %) went to a private practitioner or mission/nonprofit clinic. Of the 93 who went to an ADDO first, 28 (30 %) were referred to a public facility. The 467 individuals took a total of 581 medicines (Table 1 top), of which 128 were antimicrobials; 23 % of sick individuals took an antimicrobial. The majority of these antimicrobials were recommended by a doctor or nurse (61 %) and 32 % were recommended at an ADDO (Table 1 middle). However, nearly half of antimicrobials taken (48 %) were purchased at an ADDO, while only 29 % were obtained from a public health facility. Of the antimicrobials purchased at ADDOs, 26 % were recommended by a doctor or nurse, but this varied considerably by region (Table 1 bottom): 5 % in Singida, 23 % in Tanga, 29 % in Mbeya, and 56 % in Morogoro.

**Table 1:** Sources of recommendations and medicines for recent acute illnesses as reported in the household survey

	Percentage of all medicines obtained by location [95 % CI]			
Source of recommendation	Total *n* = 581	ADDO *n* = 269	Public facility *n* = 184	Private/mission/ NGO facility/ other *n* = 128
Doctor/nurse	60.0 [52.9, 66.6]	29.4 [20.1, 40.7]	99.0 [95.4, 99.8]	78.6 [63.2, 88.7]
ADDO	30.9 [24.5, 38.2]	58.8 [49.1, 67.8]	0.3 [0.0, 2.6]	8.3 [3.3, 19.5]
Self/ household member/ friend/ other	9.1 [6.2, 13.2]	11.8 [7.3, 18.6]	0.7 [0.1, 5.0]	13.1 [6.0, 26.1]
	Percentage of antimicrobials obtained by location [95 % CI]			
Source of recommendation	Total *n* = 128	ADDO *n* = 62	Public facility *n* = 37	Private/mission/ NGO facility/ other *n* = 29
Doctor/nurse	60.7 [47.1, 72.8]	25.6 [11.8, 46.8]	100.0 [., .]	86.9 [60.9, 96.6]
ADDO	32.3 [21.4, 45.4]	65.8 [45.1, 81.8]	-**	1.7 [0.2, 13.6]
Self/ household member/ friend/ other	7.1 [3.2, 15.0]	8.6 [3.6, 19.2]	-	11.3 [2.6, 37.9]
	Percentage of antimicrobials obtained in ADDO/DLDBs by region (n = 62) [95 % CI]			
Source of recommendation	Mbeya n = 24	Morogoro n = 9	Singida n = 14	Tanga n = 15
Doctor/nurse	29.0 [8.3, 64.9]	55.8 [14.3, 90.5]	5.1 [0.9, 23.8]	22.6 [7.8, 50.2]
ADDO	62.5 [28.8, 87.3]	44.2 [9.5, 85.7]	83.4 [49.0, 96.3]	67.1 [45.2, 83.5]
Self/ household member/ friend/ other	8.5 [2.0, 29.4]	0	11.5 [2.1, 44.4]	10.3 [2.5, 34.1]

^a^ *ADDO* accredited drug dispensing outlet

^b^All ADDO recommended antimicrobials were obtained from ADDOs

A majority of the 1185 respondents agreed that they can trust both the medicine quality (67 %) in ADDOs and the advice about treatment from the dispenser (66 %) (Additional file 1: Annex 1). They also felt that ADDOs have good quality of service (56 %), and usually have the medicines (57 %) or specifically antimicrobials (48 %) needed. This contrasted with their attitudes towards public health facilities: although half (51 %) of respondents thought the quality of care was good in PHFs, most were dissatisfied with the waiting time (66 %) or thought it likely that the medicines they needed would not be in stock there (56 %).

Respondents from the Morogoro region were more likely to have a positive opinion of ADDO services (odds ratio: 1.61 [95 % confidence intervals: 1.24, 2.08], *p* < 0.001) and to prefer ADDOs to other facilities than those from other regions (1.38 [1.24, 1.53], *p* < 0.001) (Table 2). Respondents living close to an ADDO were also more likely to prefer ADDOs to other facilities (1.12 [1.04, 1.24], *p* < 0.05). Men were more likely than women to have a negative opinion about services in public health facilities (0.82 [0.72, 0.92], *p* < 0.01). Finally, lower education was a strong predictor of positive opinion about public health facilities (1.55 [1.36, 1.78], *p* < 0.001) and ADDOS (1.31 [1.03, 1.66], *p* < 0.05), and of negative opinion about medicines affordability (0.89 [0.82, 0.95], *p* < 0.01).

**Table 2:** Predictors of summary factor scores of respondent opinions about health services from multivariate logistic regressions

		Outcomes odds ratios [95 % confidence intervals]			
Predictors	n	Services in ADDOs	Services in public health facilities	Services in ADDOs vs. other facilities	Medicine affordability
Region (vs. Mbeya)					
Morogoro	284	1.61**** [1.24,2.08]	1.13 [0.93,1.38]	1.38**** [1.24,1.53]	1.02 [0.94,1.11]
Singida	299	0.94 [0.71,1.24]	1.20* [0.97,1.48]	0.97 [0.86,1.09]	1.02 [0.93,1.12]
Tanga	300	0.81 [0.59,1.10]	0.96 [0.78,1.17]	1.04 [0.91,1.19]	0.96 [0.88,1.05]
Distance from ADDO					
< 5 km (vs. 5 km and over)	844	1.13 [0.97,1.32]	1.03 [0.86,1.23]	1.12** [1.01,1.24]	1.05 [0.98,1.12]
Household size (vs. median size)					
< median household size	468	1.01 [0.86,1.18]	1.01 [0.81,1.26]	0.95 [0.83,1.09]	1.02 [0.94,1.11]
> median household size	499	0.97 [0.83,1.12]	1.02 [0.87,1.19]	0.94 [0.84,1.06]	0.95 [0.87,1.05]
Respondent					
Male (vs. female)	583	1.08 [0.94,1.24]	0.82*** [0.72,0.92]	1.02 [0.91,1.13]	0.96 [0.90,1.01]
Age (vs. <25 years)					
25-50 years	702	0.96 [0.73,1.25]	0.81* [0.65,1.01]	1.05 [0.90,1.23]	0.93* [0.85,1.01]
> 50 years	386	1.01 [0.75,1.37]	0.87 [0.70,1.08]	1.03 [0.88,1.20]	0.90* [0.81,1.01]
No secondary school education (vs. at least some secondary school education)	1009	1.31** [1.03,1.66]	1.55**** [1.36,1.78]	0.99 [0.88,1.10]	0.89*** [0.82,0.95]

This table shows predictors of summary factor scores of respondent opinions about health services delivered in ADDOs, in public healthcare facilities, in other healthcare facilities, and medicines affordability from multivariate logistic regressions

* *p* < 0.10, ** *p* < 0.05, *** *p* < 0.01, **** *p* < 0.001

Although widely used, antimicrobials were not widely known or understood. Only 400 (34 %) of the 1185 respondents were familiar with the Kiswahili term for “antimicrobial” and only 91 (8 %) could spontaneously name one antimicrobial correctly. Slightly more respondents (9 %) spontaneously named one incorrectly, while most did not offer any name. Of the 400 respondents who said they had heard of antimicrobials, 84 % thought they were good for coughs and colds, 79 % for malaria, 68 % for diarrhea with or without blood, 63 % for pneumonia, 54 % for sexually transmitted diseases, and 46 % for purulent earache.

Of 1185 households visited, 165 households (14 %) had 215 packets of antimicrobials in their household stock, a much higher number than the 128 antimicrobials needed to treat recent acute illnesses. Indeed, respondents reported that 36 % of these antimicrobials were for current treatment and 64 % had been left over from a previous illness. Similar to reported treatment for recent illness, 61 % of these antimicrobials were recommended by a doctor or nurse and 21 % by an ADDO or drug store dispenser. However, 53 % of the antimicrobials were purchased in ADDOs, 32 % came from PHFs, and 15 % came from other sources (data not shown). Overall, 97 % of the 215 different packets of antimicrobials found at home were on the Tanzania essential medicines list.

### Mystery shoppers at ADDOs

The three scenarios presented by mystery shoppers elicited distinctly different patterns of treatment by ADDO dispensers. For the mild ARI scenario, 45 % of attendants probed for danger signs, particularly about the seriousness of the fever (25 %), 36 % asked about the duration of the illness, and 17 % about the affordability of the medicine. Only one in five attendants (22 %) offered a diagnosis to the mystery shopper; 93 % dispensed medicine (34 % antimicrobials) with 29 % giving instructions on how to take the medicine.

In comparison, with the direct request for co-trimoxazole, dispensers were half as likely to probe for danger signs, almost 14 times as likely to dispense antimicrobials, and less likely offer a diagnosis or give reasons for dispensing the medicine or instructions on how to use the medicines (Table 3).

**Table 3:** Comparing ADDO dispenser behaviors by scenario during mystery shopper visits using multivariate logistic regression models

	Odds ratio [95 % confidence intervals]	
Behavior of ADDO dispenser	Antimicrobial request scenario (*n* = 102)^a^	Pneumonia scenario (*n* = 102)^a^
Probing for danger signs	0.51** [0.28,0.94]	1.51 [0.85,2.68]
Giving a diagnosis	0.67 [0.33,1.37]	5.75**** [3.08,10.72]
Dispensing medicines	1.00 [0.33,3.07]	0.17*** [0.07,0.42]
Dispensing antimicrobial	13.67**** [6.52,28.66]	2.25*** [1.27,3.97]
Giving reasons for dispensing medicine(s)	0.73 [0.41,1.31]	1.22 [0.66,2.26]
Giving instructions on how to use medicine(s)	0.87 [0.60,1.27]	2.08**** [1.49,2.91]
Advising caregiver to watch for danger signs	0.34* [0.12,1.01]	2.47*** [1.25,4.87]
Instructing caregiver to go to the health facility	0.65 [0.26,1.61]	5.90**** [2.91,11.96]
Referring child to health facility or doctor	1.53 [0.80,2.92]	2.27** [1.14,4.51]

^a^Compared to mild ARI scenario (*n* = 102) and adjusted for region

* *p* < 0.10, ** *p* < 0.05, *** *p* < 0.01, **** *p* < 0.001

For the pneumonia scenario, the dispenser was almost six times as likely to offer a diagnosis and six times as likely to tell the caretaker to go to a health facility. He or she was more than twice as likely to dispense antimicrobials, to give instructions on how to take the medicine, and to advise the caretaker to look for danger signs and to refer the child, but overall much less likely to dispense a medicine that is not an antimicrobial (*p* < 0.001) (Table 3).

**Fig. 1:**
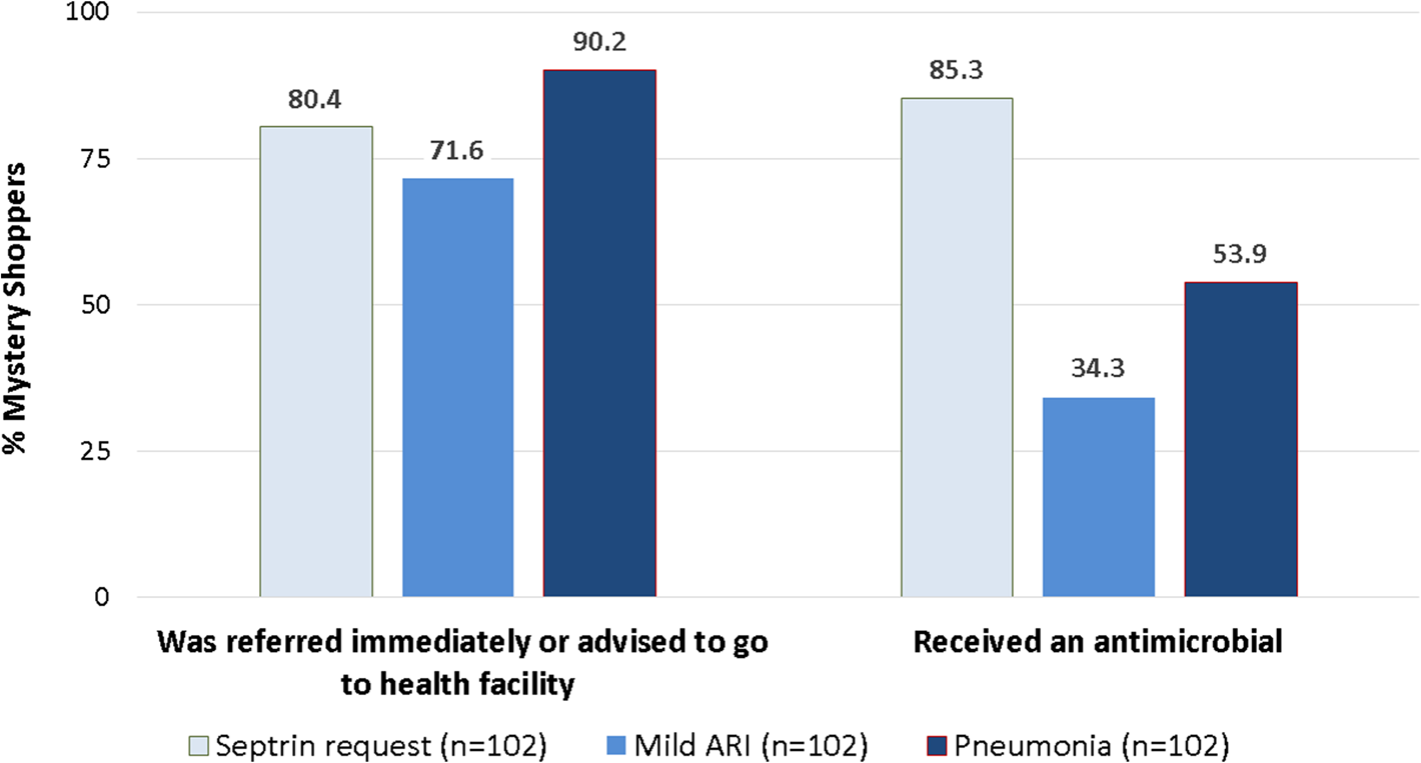
Referrals to seek health care and antimicrobial sales during mystery shopper visits by scenario

In terms of antimicrobial treatment, 34 % of mystery shoppers presenting a mild ARI were sold an antimicrobial, compared with 85 % presenting the same symptoms plus requesting an antimicrobial (Fig. 1). For customers describing symptoms of pneumonia, 54 % were sold an antimicrobial and 90 % were referred, half with an antimicrobial dispensed as well and half without. Only 1 % of mystery shoppers was neither referred nor given an antimicrobial.

### Antimicrobial availability and price in ADDOs and health facilities

Availability of antimicrobials tended to be better in ADDOs than in public health facilities, particularly for pediatric suspensions and syrups (Table 4). The average availability of antimicrobials was 63 % in ADDOs compared with 60 % in PHFs, but for suspensions the average availability was 83 % in ADDOs and 51 % in PHFs. Table 5 shows some regional differences, however. The Morogoro ADDOs had 70 % of all antimicrobials available compared to the other regions (range 62–64 %); whereas, Mbeya public health facilities had 67 % available compared to the other regions (range 57–60 %). Mbeya and Morogoro ADDOs had 89 and 90 % of suspensions available compared to the other regions’ 76 % availability, while Mbeya PHFs had 62 % of suspensions available and Singida had 42 % compared to the other regions (50 and 53 %). None of the PHFs had all the antimicrobials surveyed and 22 % had all four suspensions available, whereas, 5 % of ADDOs had all antimicrobials and 61 % had all four suspensions (Table 4).

**Table 4:** Percent availability and median MPRs of different antimicrobials by type of health care facility

	ADDOs *n* = 94		Public health care facilities *n* = 72		Private pharmacies *n* = 13	
	% Availability [95 % CI]	Median MPR (25th, 75th percentiles)^a, b^	% Availability [95 % CI]	Median MPR (25th, 75th percentiles)^a, b^	% Availability [95 % CI]	Median MPR (25th, 75th percentiles)^a^
All antimicrobials	5.3 [2.2, 12.3]		0.0 [., .]		61.5 [31.0, 85.1]	
All suspension formulations	60.6 [48.7, 71.4]		22.2 [13.4, 34.5]		76.9 [46.5, 92.8]	
Amoxicillin trihydrate 250 mg caps	81.9 [72.6, 88.6]	2.25 (1.69, 2.70)	86.1 [74.9, 92.8]	1.69 (1.35, 2.70)	100.0 [., .]	1.69 (1.69, 2.36)
Amoxicillin trihydrate 125 mg/5 mL suspension	86.2 [77.0, 92.1]	1.96 (1.84, 2.45)	62.0 [49.2, 73.3]	1.84 (1.10, 2.45)	100.0 [., .]	1.84 (1.22, 2.45)
Co-trimoxazole 480 mg tablets	87.2 [78.9, 92.6]	2.65 (2.12, 3.19)	84.7 [73.9, 91.6]	1.86 (1.33, 2.65)	92.3 [59.7, 99.0]	2.65 (2.39, 2.65)
Co-trimoxazole 240 mg/5 mL suspension	89.4 [81.3, 94.2]	1.80 (1.80, 2.40)	60.6 [48.0, 71.9]	1.80 (1.13, 2.22)	92.3 [59.7, 99.0]	1.80 (1.20, 2.40)
Erythromycin 250 mg tablets	75.5 [64.8, 83.8]	1.59 (1.32, 1.59)	83.1 [69.7, 91.3]	1.30 (0.63, 1.59)	100.0 [., .]	1.59 (1.59, 1.59)
Erythromycin 125 mg/5 mL suspension	74.5 [63.2, 83.2]	1.33 (1.33, 1.67)	54.2 [41.4, 66.4]	1.33 (1.21, 1.73)	92.3 [59.7, 99.0]	1.33 (1.33, 1.50)
Metronidazole 200 mg tablets	90.4 [83.0, 94.8]	3.28 (2.95, 4.92)	93.1 [84.0, 97.2]	2.46 (1.64, 3.93)	92.3 [59.7, 99.0]	3.11 (1.97, 4.92)
Metronidazole 200 mg/5 mL suspension	80.9 [71.0, 87.9]	3.24 (2.43, 3.24)	29.2 [19.2, 41.7]	2.43 (1.70, 3.24)	92.3 [59.7, 99.0]	2.43 (2.19, 3.24)
Ampicillin 250 mg caps	29.8 [21.3, 40.0]	2.14 (1.60, 2.25)	7.0 [2.9, 16.0]	2.57 (1.60, 3.08)	69.2 [42.7, 87.2]	1.60 (1.28, 1.60)
Ciprofloxacin 500 mg tablets	36.2 [26.9, 46.6]	2.38 (1.59, 3.17)	76.4 [63.2, 85.9]	2.38 (1.59, 3.02)	100.0 [., .]	1.59 (1.59, 2.38)
Ampicillin/ cloxacillin 500 mg caps	31.9 [22.9, 42.5]	Not available	23.6 [14.8, 35.5]	Not available	100.0 [., .]	Not available
Doxycycline 100 mg caps/tablets	60.6 [50.6, 69.8]	4.17 (3.33, 4.17)	86.1 [75.1, 92.7]	4.17 (2.08, 4.17)	100.0 [., .]	4.17 (2.08, 4.17)
Tetracycline 250 mg caps	28.3 [19.9, 38.4]	2.26 (2.26, 2.63)	8.3 [3.7, 17.8]	2.26 (0.90, 2.26)	92.3 [59.7, 99.0]	2.26 (2.03, 2.26)
Procaine penicillin fortified 4MU, powder for injection	52.1 [41.1, 63.0]	2.23 (1.78, 2.23)	62.5 [51.2, 72.6]	2.01 (0.72, 2.79)	76.9 [49.9, 91.8]	1.78 (1.56, 2.23)
Benzyl penicillin 5MU, powder for injection	44.7 [33.3, 56.6]	2.44 (2.19, 2.44)	88.9 [78.3, 94.7]	2.44 (1.22, 3.05)	84.6 [57.2, 95.8]	1.95 (1.71, 2.44)

This table shows the percent availability and median prices in relation to international reference prices of different antimicrobials by type of health care facility

^a^ Median price ratio (MPR) = median unit price in outlets/*International Drug Price Indicator Guide* reference procurement price

^b^ Comparison of the 14 available antimicrobials median MPRs between ADDOs and public health care facilities: *p* = 0.0425 (paired *t*-test)

**Table 5:** Average availability of all antimicrobials and of antimicrobial suspension formulations by facility type and region

	ADDOs mean percent available (SD)	Public health facilities mean percent available (SD)
All antimicrobials		
All regions	63.26 % (20.03 %)	60.19 % (20.17 %)
Mbeya	62.22 % (18.09 %)	67.08 % (14.50 %)
Morogoro	70.28 % (12.74 %)	57.25 % (23.22 %)
Singida	56.81 % (27.55 %)	57.46 % (22.06 %)
Tanga	63.48 % (18.08 %)	60.00 % (19.27 %)
Suspension formulations		
All regions	82.71 % (26.19 %)	51.04 % (36.68 %)
Mbeya	88.54 % (22.09 %)	62.50 % (30.28 %)
Morogoro	89.58 % (24.36 %)	50.00 % (37.50 %)
Singida	76.09 % (26.63 %)	41.67 % (44.25 %)
Tanga	76.09 % (29.66 %)	52.78 % (30.78 %)

**Fig. 2:**
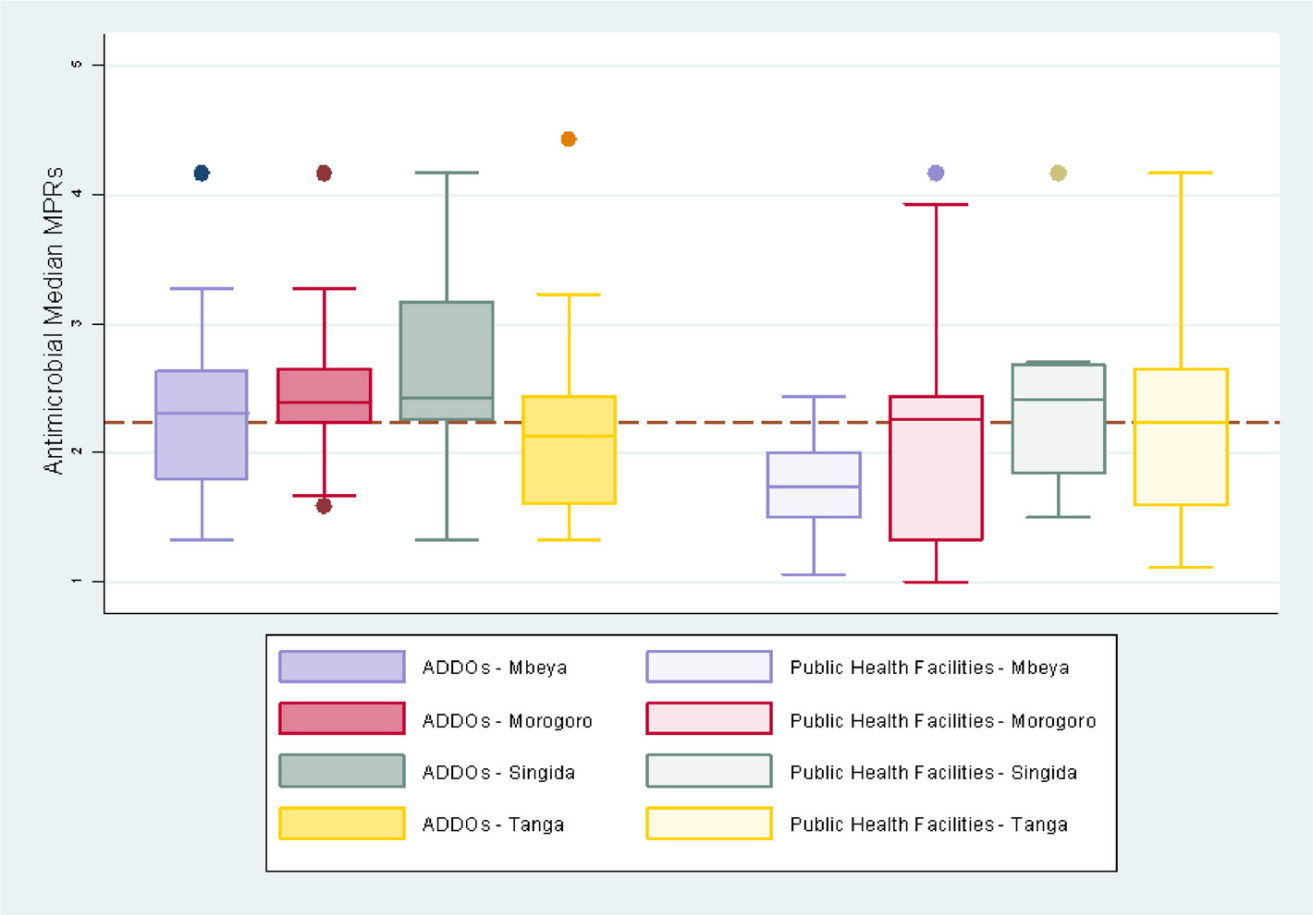
Regional median of median price ratios MPRs* for antimicrobials in ADDOs and public health facilities

Table 4 shows the MPR for each individual antimicrobial and Table 6 shows the MPR for antimicrobials overall which was in ADDOs 2.42, compared with 2.16 for PHFs, which indicated that antimicrobials were on average 12 % more expensive in ADDOS than in PHFs. For suspensions only, the price ratio compared to the median reference price in ADDOs was 2.12 and 1.89 in PHFs, again indicating that prices in ADDOs were about 12 % more expensive. However, there were regional differences (Table 6 and Fig. 2); in Tanga, PHFs’ antimicrobials were 4 % more expensive than in ADDOs, whereas, antimicrobials in ADDOs were 12 % more expensive than in PHFs in Singida, 16 % more in Morogoro, and 30 % more in Mbeya. For suspensions, ADDOs were 13 % less costly than they were in PHFs in Tanga and 1 % less in Mbeya, but suspensions were 14 % more expensive in Singida ADDOs and 60 % more in Morogoro ADDOs.

**Table 6:** Mean of antimicrobial MPRs^a^ for antimicrobials in ADDOs and public health facilities

	All Antimicrobials			Suspensions		
Region	ADDO MPR	PHF MPR	Ratio ADDO to PHF	ADDO MPR	PHF MPR	Ratio ADDO to PHF
Mbeya	2.32	1.79	1.30	1.85	1.86	0.99
Morogoro	2.51	2.17	1.16	2.44	1.52	1.60
Singida	2.62	2.33	1.12	2.36	2.06	1.14
Tanga	2.25	2.33	0.96	1.85	2.13	0.87
All	2.42	2.16	1.12	2.12	1.89	1.12

^a^ Median price ratio (MPR) = median unit price in outlets/*International Drug Price Indicator Guide* reference procurement price. PHF = public health facilities

This table shows the mean of medicine price ratios (MPRs) of all antimicrobials and of antimicrobial pediatric formulations in ADDOs and public health facilities, overall and by region

Detailed antimicrobial dispensing records were available in only 47 of the 94 ADDOs. In 233 instances of antimicrobial dispensing in these ADDOs, 63 % of antimicrobials were dispensed on prescription rather than being recommended by the ADDO dispenser.

## Discussion

From the household survey, we have seen that clinicians were the main cadre to recommend antimicrobials, but more antimicrobials were purchased at ADDOs than PHFs. This suggests that people value the advice of public sector health professionals, but buy their medicines mostly at ADDOs either because of preference or supply system failures at PHFs. Consumers largely see ADDOs as convenient, well-stocked—especially with antimicrobials—and as having good quality advice and products. This is true even though prices of tracer medicines averaged 12 % more than in PHFs (although this varied by region). Availability of antimicrobials was also better in ADDOs, especially for children’s formulations. Given that PHFs are intended to sell medicines procured through public systems with minimal price mark-up, the ability of ADDOs to be price-competitive is somewhat surprising. The convenience of a local retail drug shop and relative certainty of availability may outweigh the time and cost of visiting a PHF for many people in the community.

Overall, people in rural Tanzania clearly think of ADDOs as part of their health system, with the most positive opinions expressed in Morogoro, where of the four sample regions, ADDOs have been in operation for the longest time. The private sector is an important source of care for the poor in low- and middle-income countries a review of data from 38 countries indicated [17]. The current study is interesting because it showed how the community combines its use of the public and private sectors, which confirms an earlier study on malaria treatment [13]; moreover, it confirms that the accreditation program in Tanzania has succeeded in the intent described in its development [10].

The findings from the mystery shopper ADDO encounters illustrated that antimicrobials are still overused in the community; for example, ADDO dispensers nearly always supplied an antimicrobial on direct request (80 %), which is more than the 49 % an earlier investigation reported [18]. However, dispensers sell fewer antimicrobials when customers ask for their opinion on the same symptoms (34 %). This is an encouraging sign of improvement since a 2008 assessment in which ADDO dispensers sold antimicrobials to 76 % of caretakers of a one year-old child with watery diarrhea [12]. Another positive sign is that only 23 % of people with an acute illness seeking care were dispensed an antimicrobial, compared to an average of 36 % in five national household surveys in Africa [19]. What is the most reassuring is that ADDO dispensers treated pneumonia symptoms more seriously and appropriately, which is a considerable improvement over other studies’ results where dispensers had little ability to manage respiratory conditions appropriately [20]. The fact that dispensers sold the majority (63 %) of antimicrobials based on a PHF prescription means that antimicrobial overuse is not just an ADDO problem, but also a public health sector problem that is exacerbated by gaps in consumer knowledge. Only one-third of household respondents said they had heard of antimicrobials and most did not know about their appropriate use.

This study has several limitations. Our four regions may not represent the whole of Tanzania. The household survey depended on reporting that is subject to recall bias. The ADDO dispensers may not have seen mystery shoppers as normal customers and therefore treated them differently. Our research would have been stronger if we had compared prescribing practices for similar patients between ADDOs and public health facilities, but most ADDOs lack records on diagnoses and prescribing. However, from the limited sample of ADDO dispensing records examined, of which 63 % of antimicrobials were dispensed on prescription, it is likely that many dispensing habits of dispensers mirror PHF prescriptions. Although all dispensers had received ADDO training, some retail drug shops in our study regions were awaiting official accreditation. Because household members could not reliably distinguish between accredited shops and those awaiting accreditation, we combined them under the “ADDO” label for analysis, which may have underestimated the ADDOs’ positive effect. In the mystery shopper and price and availability surveys, however, we only visited fully accredited shops.

Although most treatment advice appears to originate from doctors and nurses, and they prescribed most of the medicines dispensed in ADDOs, serious problems remain. Community members know little about antimicrobials and their recommended use. In addition, ADDO dispensers readily sell antimicrobials on request despite being trained not to do so and realizing that the practice is incorrect [21].

Successful interventions to improve community use of antimicrobials require a combination of approaches targeting all stakeholders, including not only ADDO dispensers but also community members, public sector prescribers, and government regulators [22]. An earlier study in Tanzania showed that a public awareness campaign using posters, specific training of ADDO dispensers, and education of public facility staff improved the use of antimicrobials [23]. Interventions in the private sector may be effective [24], in particular changing incentives and accountability may be the most effective way of changing behavior [25]. The WHO strongly recommends monitoring antimicrobial use as a way to control antimicrobial resistance [26]. Initiating active supervision of antimicrobial dispensing practices in ADDOs and linking the results to accreditation status may comprise an effective strategy when coupled with similar monitoring in PHFs and with public campaigns to counteract inadequate community knowledge of antimicrobials.

## Conclusion

Accredited drug dispensing outlets have increased rural Tanzanian’s access to antimicrobials and are viewed as an integral part of the health care system. Use of antimicrobials remains suboptimal, however, but this stems not only from poor dispensing practices in ADDOs, but also from poor prescribing and antimicrobial availability in public facilities as well as inappropriate consumer demand rooted in poor understanding. To improve how the community uses antimicrobials, multi-pronged interventions to increase appropriate practices in both ADDOs and public health facilities need to be combined with active monitoring of those practices.

## Additional file

Additional file 1:
**Annex 1.** Household survey respondent opinions about health services and medicines. Annex 2. Predictors of summary factor scores of respondent opinions about health services from univariate logistic regressions. (DOCX 45 kb)

## References

[1] World Health Organization (2014). Antimicrobial resistance: global report on surveillance 2014.

[2] World Health Organization Patient Safety Programme (2012). The evolving threat of antimicrobial resistance: options for action.

[3] World Health Organization (2011). The world medicines situation 2011, rational use of medicines.

[4] Berman P (2000). Organization of ambulatory care provision: a critical determinant of health system performance in developing countries. Bull World Health Organ.

[5] McCombie SC (2002). Self-treatment for malaria: the evidence and methodological issues. Health Policy Plan.

[6] Goodman C, Kachur SP, Abdulla S, Bloland P, Mills A (2007). Drug shop regulation and malaria treatment in Tanzania: why do shops break the rules, and does it matter?. Health Policy Plan.

[7] Brieger WR, Unwin A, Greer G, Meek S. Interventions to improve the role of informal private providers in malaria case management for children in Africa. BASICS II and the Malaria Consortium; prepared for The Malaria Case Management Working Group, Roll Back Malaria; 2004. www.rollbackmalaria.org/files/files/partnership/wg/wg_management/docs/medsellersRBMmtgsubcommitteereport.pdf. Accessed 1 Dec 2014.

[8] Williams HA, Jones CO (2004). A critical review of behavioral issues related to malaria control in sub-Saharan Africa: what contributions have social scientists made?. Soc Sci Med.

[9] Wagner AK, Graves AJ, Reiss SK, Le Cates R, Zhang F, Ross-Degnan D (2011). Access to care and medicines, burden of health care expenditures, and risk protection: results from the world health survey. Health Policy.

[10] Center for Pharmaceutical Management (2003). Access to essential medicines: Tanzania, 2001. Prepared for the Strategies for Enhancing Access to Medicines Program.

[11] Rutta E, Senauer K, Johnson K, Adeya G, Mbwasi R, Liana J (2009). Creating a new class of pharmaceutical services provider for underserved areas: the Tanzania accredited drug dispensing outlet experience. Prog Community Health Partnersh.

[12] EADSI (East African Drug Seller Initiative) (2011). Evaluation report.

[13] Alba S, Hetzel MW, Goodman C, Dillip A, Liana J, Mshinda H (2010). Improvements in access to malaria treatment in Tanzania after switch to artemisinin combination therapy and the introduction of accredited drug dispensing outlets - a provider perspective. Malar J.

[14] World Health Organization and Medicines Transparency Alliance. Household & facility surveys on access to and rational use of medicines in countries. http://www.medicinestransparency.org/meta-toolbox/household-facility-surveys-on-access-to-and-rational-use-of-medicines-in-countries/. Accessed on 28 Jun 2015.

[15] Madden JM, Quick JD, Ross-Degnan D, Kafle KK (1997). Undercover careseekers; simulated clients in the study of health provider behaviour in developing countries. Soc Sci Med.

[16] Management Sciences for Health (2013). International drug price indicator guide, 2012 edition.

[17] Bustreo F, Harding A, Axelsson H (2003). Can developing countries achieve adequate improvements in child health outcomes without engaging the private sector?. Bull World Health Organ.

[18] Kagashe GA, Minzi O, Matowe L (2011). An assessment of dispensing practices in private pharmacies in Dar-es-Salaam, Tanzania. Int J Pharm Pract.

[19] Vialle-Valentin CE, Lecates RF, Zhang F, Desta AT, Ross-Degnan D (2012). Predictors of antibiotic use in African communities: evidence from medicines household surveys in five countries. Trop Med Int Health.

[20] Tumwikirize WA, Ekwaru PJ, Mohammed K, Ogwal-Okeng JW, Aupont O. Management of acute respiratory infections in drug shops and private pharmacies in Uganda: a study of counter attendants' knowledge and reported behaviour. East Afr Med J. 2004;Suppl Feb:S33–40. 15125114

[21] Dillip A, Embrey M, Shekalaghe E, Ross-Degnan D, Vialle-Valentin C, Kimatta S, Liana J, Rutta E, Valimba R, Chalker J (2015). What motivates antibiotic dispensing in accredited drug dispensing outlets in Tanzania? A qualitative study. Antimicrob Resist Infect Control.

[22] Goodman C, Brieger W, Unwin A, Mills A, Meek S, Greer G (2007). Medicine sellers and malaria treatment in sub-Saharan Africa: what do they do and how can their practice be improved?. Am J Trop Med Hyg.

[23] Valimba R, Liana J, Joshi MP, Rutta E, Embrey M, Bundala M, Kibassa B. Engaging the private sector to improve antimicrobial use in the community: experience from accredited drug dispensing outlets in Tanzania. J Pharm Policy Pract. 2014; doi:10.1186/2052-3211-7-1.1 10.1186/2052-3211-7-11 PMC4177420 25298887

[24] Wafula FN, Goodman CA (2010). Are interventions for improving the quality of services provided by specialized drug shops effective in sub-Saharan Africa? A systematic review of the literature. Qual Assur Health Care.

[25] Shah NM, Brieger WR, Peters DH (2010). Can interventions improve health services from informal private providers in low and middle-income countries? A comprehensive review of the literature. Health Policy Plan.

[26] World Health Organization (2001). Global strategy for containment of antimicrobial resistance.

