# Supplementary material for: What roles do accredited drug dispensing outlets in Tanzania play in facilitating access to antimicrobials? Results of a multi-method analysis
Source: Antimicrob Resist Infect Control. 2015 Aug 20;4:33. doi: 10.1186/s13756-015-0075-2 (PMC4545914; doi:10.1186/s13756-015-0075-2)
Supplement: Additional file 1: — Annex 1. Household survey respondent opinions about health services and medicines. Annex 2. Predictors of summary factor scores of respondent opinions about health services from univariate logistic regressions. (DOCX 45 kb) [file 13756_2015_75_MOESM1_ESM.docx]

**Annex 1. Household survey respondent opinions about health services and medicines**

|  | N | Agree Proportion [95% CI] | Disagree Proportion [95% CI] | Don't know Proportion [95% CI] |
| --- | --- | --- | --- | --- |
| 1 - Services in ADDOs |  |  |  |  |
| When a dispenser in an ADDO recommends a medicine, you can be sure it is of good quality | 1182 | 0.67  [0.6, 0.74] | 0.23  [0.18, 0.29] | 0.1  [0.06, 0.15] |
| You trust the ADDO dispenser to give the right advice about treatment | 1180 | 0.66  [0.59, 0.73] | 0.25  [0.2, 0.29] | 0.09  [0.06, 0.14] |
| The quality of services is good at ADDOs in your community | 1185 | 0.56  [0.5, 0.63] | 0.32  [0.27, 0.37] | 0.12  [0.09, 0.16] |
| The ADDO closest to your household usually has the medicines you need | 1181 | 0.57  [0.51, 0.62] | 0.29  [0.25, 0.34] | 0.14  [0.11, 0.18] |
| You can always obtain antibiotics at the ADDO when you need them | 1179 | 0.48  [0.41, 0.55] | 0.15  [0.13, 0.18] | 0.37  [0.31, 0.44] |
| When ADDO dispensers recommends a medicine, you can be sure it is best value for money | 1167 | 0.39  [0.34, 0.45] | 0.37  [0.33, 0.41] | 0.24  [0.19, 0.29] |
| ADDO dispensers take into account your ability to pay when deciding which medicines to sell | 1162 | 0.37  [0.33, 0.42] | 0.47  [0.42, 0.51] | 0.16  [0.12, 0.21] |
| Your household can usually get credit from the ADDO if you need it | 1166 | 0.21  [0.17, 0.26] | 0.7  [0.65, 0.75] | 0.09  [0.06, 0.15] |
| 2 - Services in Public Health Facilities |  |  |  |  |
| The quality of services is good at the public health care facility where you usually seek care | 1183 | 0.51  [0.46, 0.56] | 0.48  [0.42, 0.53] | 0.01  [0.01, 0.02] |
| The public health care facility where you usually seek care has the medicines you need | 1182 | 0.41  [0.36, 0.46] | 0.56  [0.51, 0.61] | 0.03  [0.02, 0.04] |
| The waiting time at the public health care facility where you usually seek care is reasonable | 1175 | 0.32  [0.27, 0.38] | 0.66  [0.6, 0.71] | 0.02  [0.01, 0.03] |
| Your household can get free medicine at the public health care facility | 1184 | 0.18  [0.15, 0.21] | 0.82  [0.79, 0.84] | 0.01  [0, 0.01] |
| 3 - Services in ADDOs vs. Other Facilities |  |  |  |  |
| Medicines are more expensive at ADDOs than in the public health care facility | 1182 | 0.72  [0.67, 0.76] | 0.16  [0.13, 0.2] | 0.12  [0.08, 0.17] |
| ADDOs are the most convenient places to seek care in your community | 1184 | 0.62  [0.56, 0.67] | 0.25  [0.21, 0.29] | 0.14  [0.1, 0.18] |
| ADDO staff sometimes refer customers to public health facilities for care | 1182 | 0.57  [0.52, 0.62] | 0.29  [0.24, 0.35] | 0.13  [0.1, 0.18] |
| The quality of services is better in ADDOs than in ordinary drug shops | 1183 | 0.43  [0.39, 0.48] | 0.21  [0.17, 0.25] | 0.36  [0.31, 0.42] |
| 4. Medicine Affordability |  |  |  |  |
| Your household can usually afford to buy the medicines you need | 1175 | 0.56  [0.51, 0.62] | 0.42  [0.37, 0.47] | 0.02  [0.01, 0.03] |
| In the past your household has had to borrow money, or sell things to pay for medicines | 1171 | 0.48  [0.44, 0.52] | 0.52  [0.47, 0.56] | 0 |

**Annex 2. Predictors of summary factor scores of respondent opinions about health services from univariate logistic regressions**

|  |  | Outcomes Odds Ratios [95% confidence intervals]) | | | |
| --- | --- | --- | --- | --- | --- |
| Predictors | **n** | **Services in ADDOs** | **Services in Public Health Facilities** | **Services in ADDOs vs. Other Facilities** | **Medicines Affordability** |
| Region (vs. Mbeya) |  |  |  |  |  |
| Morogoro | 284 | 1.60** [1.20,2.12] | 1.07 [0.82,1.40] | 1.38*** [1.24,1.53] | 1.02 [0.93,1.11] |
| Singida | 299 | 0.91 [0.70,1.19] | 1.21 [0.96,1.53] | 0.95 [0.85,1.06] | 1.00 [0.91,1.10] |
| Tanga | 300 | 0.80 [0.59,1.08] | 0.98 [0.77,1.23] | 1.03 [0.91,1.18] | 0.97 [0.88,1.07] |
| Distance from ADDO |  |  |  |  |  |
| <5 km (vs. 5 km and over) | 844 | 1.15 [0.96,1.37] | 0.98 [0.82,1.17] | 1.16** [1.04,1.30] | 1.07* [1.00,1.15] |
| Household size (vs. median size) |  |  |  |  |  |
| < median HH size | 468 | 1.01 [0.88,1.16] | 1.02 [0.80,1.30] | 0.95 [0.83,1.09] | 1.02 [0.93,1.11] |
| > median HH size | 499 | 0.95 [0.82,1.10] | 1.03 [0.88,1.22] | 0.94 [0.82,1.06] | 0.94 [0.86,1.04] |
| Respondent |  |  |  |  |  |
| Male (vs. female) | 583 | 1.14 [0.97,1.34] | 0.80** [0.70,0.92] | 1.04 [0.94,1.15] | 0.95^+^ [0.91,1.00] |
| Age (vs. <25 years ) |  |  |  |  |  |
| 25-50 years | 702 | 1.07 [0.84,1.37] | 0.86 [0.69,1.06] | 1.09 [0.92,1.27] | 0.90* [0.82,0.99] |
| >50 years | 386 | 1.18 [0.88,1.59] | 0.88 [0.70,1.10] | 1.07 [0.93,1.25] | 0.86** [0.78,0.96] |
| No secondary school education (vs. at least some secondary school education) | 1009 | 1.20* [1.02,1.42] | 1.56*** [1.36,1.80] | 0.94 [0.86,1.03] | 0.88** [0.81,0.95] |

^+^ p<0.10, * p<0.05, ** p<0.01, *** p<0.001

This table shows the predictors of summary factor scores of respondent opinions about health services delivered in ADDOs, in public healthcare facilities, in other healthcare facilities, and medicines affordability from univariate logistic regressions
